# The Effects of Exercise Intensity *vs*. Metabolic State on the Variability and Magnitude of Left Ventricular Twist Mechanics during Exercise

**DOI:** 10.1371/journal.pone.0154065

**Published:** 2016-04-21

**Authors:** Craig Armstrong, Jake Samuel, Andrew Yarlett, Stephen-Mark Cooper, Mike Stembridge, Eric J. Stöhr

**Affiliations:** Discipline of Physiology & Health, Cardiff Metropolitan University, Cardiff, Wales, United Kingdom; Scuola Superiore Sant'Anna, ITALY

## Abstract

Increased left ventricular (LV) twist and untwisting rate (LV twist mechanics) are essential responses of the heart to exercise. However, previously a large variability in LV twist mechanics during exercise has been observed, which complicates the interpretation of results. This study aimed to determine some of the physiological sources of variability in LV twist mechanics during exercise. Sixteen healthy males (age: 22 ± 4 years, $\dot{V}$O_2peak_: 45.5 ± 6.9 ml∙kg^-1^∙min^-1^, range of individual anaerobic threshold (IAT): 32–69% of $\dot{V}$O_2peak_) were assessed at rest and during exercise at: i) the same relative exercise intensity, 40%_peak_, ii) at 2% above IAT, and, iii) at 40%_peak_ with hypoxia (40%_peak_+HYP). LV volumes were not significantly different between exercise conditions (*P* > 0.05). However, the mean margin of error of LV twist was significantly lower (*F*_2,47_ = 2.08, *P* < 0.05) during 40%peak compared with IAT (3.0 *vs*. 4.1 degrees). Despite the same workload and similar LV volumes, hypoxia increased LV twist and untwisting rate (*P* < 0.05), but the mean margin of error remained similar to that during 40%peak (3.2 degrees, *P* > 0.05). Overall, LV twist mechanics were linearly related to rate pressure product. During exercise, the intra-individual variability of LV twist mechanics is smaller at the same relative exercise intensity compared with IAT. However, the absolute magnitude (degrees) of LV twist mechanics appears to be associated with the prevailing rate pressure product. Exercise tests that evaluate LV twist mechanics should be standardised by relative exercise intensity and rate pressure product be taken into account when interpreting results.

## Introduction

Performing exercise requires complex cardiovascular adjustments to meet the specific peripheral and central energetic demands. The contribution of the heart in generating the necessary amount of blood flow during exercise is well-described and is undisputed. For example, cardiac output increases substantially from rest to near-maximal exercise intensities and the maximally achievable cardiac output is a major determinant of human exercise capacity as expressed in the classic Fick equation [1,2]. However, how the human heart muscle generates the cardiac output during exercise remains poorly understood. Recent studies have highlighted that left ventricular twist and untwisting rate (LV twist mechanics) are essential to cardiac function during exercise. Clinical reports show that the increase in LV twist mechanics during exercise in healthy individuals [3,4,5,6] is clearly blunted in patients with cardiac dysfunction [7,8,9,10]. These studies indicate that the magnitude of LV twist mechanics during exercise is an important discriminator between normal and reduced cardiac performance. However, previous studies have also reported a relatively large inter-individual variability in LV twist mechanics during exercise, with a mean margin of error of LV twist of 2.3 degrees at rest (99% CI = 8.7 to 13.3 degrees), which doubled to 4.6 degrees during exercise (99% CI = 19.4 to 28.6 degrees; data based on reference [7]; Estimates are based upon the equation: $99\%\;\text{CI}\;=\bar{x}\pm \left(\frac{2.58\;\sigma }{\sqrt{n}}\right)$, where $\bar{x}$ is the mean of the sample, *σ* the standard deviation and $\sqrt{n}$ the square root of the sample size). Even more pronounced was the threefold increase in the margin of error of LV untwisting rate from 23 degrees∙sec^-1^ at rest to 76 degrees∙sec^-1^ during exercise. The reason for this increased variability during exercise is difficult to determine because LV twist is influenced by preload, afterload and contractility and all these factors change concurrently with exercise [11,12,13,14]. Still, the investigation of cardiac deformation parameters such as LV twist mechanics is increasingly popular in the clinical and research setting and therefore attempting to improve the current knowledge of the LV twist response to exercise seems warranted [7,8,9,15]. A better understanding of the physiological determinants of the variability and magnitude of LV twist mechanics during exercise will advance our general understanding of LV twist mechanics. Improving the interpretation of exercise responses will facilitate a more accurate prescription of exercise tests for investigating LV function in the clinical and research setting. Measurement of torsion-to-shortening ratio, which has been suggested to reflect the contribution of the endocardium and epicardium to overall LV twist [16], may further clarify the sources of variability in LV twist mechanics during exercise.

Considering fundamental principles of cardiovascular exercise physiology, two hypotheses can be formulated that each provide a reasonable assumption for the determinants of the magnitude of LV twist mechanics during exercise. First, it is known that cardiac output, myocardial oxygen consumption and coronary blood flow increase *linearly* from rest to near-maximal exercise intensities [17]. These phenomena support the assumption that sub-maximal LV twist mechanics may be directly related to relative exercise intensity because a similar relative change in cardiac output and coronary blood flow from rest to near-maximal exercise will occur in all individuals. In contrast, every human possesses what is known as the ‘individual anaerobic threshold’ (IAT), which is the exercise intensity above which metabolism is markedly altered as reflected by the *exponential* rise in lactate and an excess CO_2_ production [18,19]. In contrast to the consistent relative change in cardiac output from rest to exercise, IAT differs between individuals. Since the heart muscle is responsive to changes in whole-body metabolism during exercise [20], the second hypothesis is that the magnitude of LV twist during exercise is disproportionately influenced by the IAT. By extension, if metabolic state was a greater determinant of LV twist mechanics than performing the same absolute exercise intensity in hypoxia–which is known to cause heterogeneous physiological responses [21]—should increase the variability in LV twist mechanics. At present, exercise intensities for the benefit of training effects can be prescribed as a percentage relative to maximal capacity or relative to IAT, the latter in populations who are advised to avoid maximal effort [22]. However, it remains unknown whether the different types of prescription influence LV twist mechanics. Since LV twist mechanics have been associated with LV myofibre stress [23] and LV stiffness [24], knowledge of the impact of different exercise intensities and IAT may have important implications for individuals partaking in regular exercise.

Based upon the considerations outlined above, the aim of the present study was twofold. Firstly, we aimed to determine whether the variability of LV twist mechanics during exercise is different when the same individuals exercise at similar relative exercise intensity compared with a similar individual metabolic state as determined by IAT. Secondly, we aimed to examine whether an additional metabolic challenge–in this case administration of hypoxia during the same absolute exercise intensities–would further influence the magnitude of LV twist mechanics. Although both hypotheses presented above appeared plausible, in this experiment the second hypothesis, which was related to a greater influence of individual metabolic state, was favoured because of the previous observation that young men with a higher aerobic fitness had a lower LV twist during exercise at the same relative exercise intensity [6].

## Methods

Following ethical approval from the Cardiff School of Sport Research Ethics Sub-Committee, Cardiff Metropolitan University, UK, 20 healthy males, who were non-smokers, volunteered to take part in the study. They provided written and verbal informed consent and completed the experimental protocol. Subsequently, the quality of echocardiographic images was insufficient in two individuals and other logistical errors required elimination of another two participants, resulting in a final study sample size of 16 participants (age: 22 ± 4 years; height: 176 ± 4 cm; body mass: 77 ± 10 kg, $\dot{V}$O_2peak_: 45.5 ± 6.9 ml∙kg^-1^∙min^-1^). Individuals with varied physical activity backgrounds were recruited in order to obtain a sample with a wide range of individual anaerobic thresholds (IAT), which was successfully achieved (range of IAT = 32–69% of $\dot{V}$O_2peak_). This study conformed to the standards set by the latest revision of the Declaration of Helsinki (October 2013) and procedures used were in agreement with institutional guidelines.

### Data collection

Participants attended the laboratory twice: The first visit served to determine participants’ aerobic fitness by completion of a standardised incremental $\dot{V}$O_2peak_ test. The purpose of the second visit was to collect experimental data. Since data collection was performed within the same day, no dietary restrictions were prescribed.

#### Visit 1 –Aerobic fitness ($\dot{V}$O_2peak_) test

Participants were weighed to determine body mass to the nearest 100 g. The $\dot{V}$O_2peak_ test was conducted on a supine cycle ergometer (Angio 2003, Lode, Groningen, Netherlands) with participants wearing a tightly fitting face mask that recorded breath-by-breath respiratory data into an online gas analysis system (OxyconPro, Jaeger at Viasys Healthcare, Warwick, UK). Following a brief resting period in the tilted position (45 degrees), participants started to cycle at 40 Watts for two minutes, at a cadence of 60 rpm. Exercise intensity then increased in a step-wise fashion by 40 Watts every four minutes. Peak power output and heart rate were recorded at the time point of task failure. $\dot{V}$O_2peak_ was calculated at the highest five-second average achieved during the test.

#### Visit 2 –Experimental protocol

Upon arrival at the laboratory participants were weighed before lying down on the supine cycle ergometer. Then, a small finger cuff was place around the middle finger of the right hand for beat-by-beat estimation of the brachial blood pressure waveform (FinometerPRO, Finapres, Arnhem, Netherlands), which was recorded continuously in a data capture system (PowerLab, ADInstruments, Oxford, UK). A pulse oximeter (Autocorr 3304, Smiths Medical PM inc, Waukesha, USA) was attached to the ring finger of the right hand for minute-by-minute assessment of oxygen saturation (SaO_2_) and ECG leads attached to the ultrasound system (Vivid E9, GE Vingmed Ultrasound, Horten Norway) were connected to the participants’ chest to record heart rate. Furthermore, each participant wore a face mask for the continuous recording of breath-by-breath respiratory data as described above. Once all the equipment was attached and recording, participants rested on the supine ergometer for at least 5 minutes with their legs fully extended on the bed. A blood sample (20μL) was drawn from the earlobe into a capillary tube and mixed with anti-haemolysing solution for later offline analysis of lactate (Biosen, C-Line Sport, EKF Diagnostics, Magdeburg, Germany). Then, echocardiographic images were obtained by an experienced sonographer (EJS) using a 4-D probe (4V, GE Medical Systems Israel LTD, Israel) and a commercially available ultrasound system (Vivid E9, GE Vingmed Ultrasound, Horten, Norway). Images were obtained in accordance with current guidelines [25] and as outlined in previous publications by the same research group [5,6]. Three to five cardiac cycles of the following views were recorded at brief end-expiratory breath hold: parasternal long-axis, parasternal short-axis (mitral valve and apex) and apical triplane images. Following the resting assessment, participants’ feet were strapped into the cycle ergometer. Each participant then performed three exercise bouts, separated by 15 minutes of supine rest: i) 40% maximal aerobic capacity (40%peak = same relative exercise intensity between individuals), ii) IAT (= same metabolic state between individuals), and, iii) when metabolism was manipulated during 40%peak by administration of hypoxia (40%peak+HYP, FiO_2_ = 12%). The exercise protocol was initially identical to that prescribed during visit one. Once participants had reached their target power output according to the three conditions identified above, exercise intensity was kept constant for six minutes. Continuously collected data were recorded throughout; echocardiographic images were obtained in the last two minutes of each exercise condition. The order of exercise conditions i) and ii) was determined by the absolute intensity prescribed, with the lower intensity performed first, followed by the higher intensity. To avoid a carry-over effect of the hypoxic stimulus, every participant performed condition iii) last.

#### Visit 2 –Data analysis

Echocardiography. For all echocardiographic windows, images were exported from the ultrasound and imported into a commercially available software package (EchoPAC Version 112 revision 1.0, GE Vingmed Ultrasound, Horten, Norway). Where possible, data were analysed for three consecutive cardiac cycles and the average was calculated. Left ventricular volumes (end-diastolic volume, end-systolic volume and stroke volume) were determined by manual tracing of apical triplane images at end-diastole and end-systole, as per the software manufacturer’s guidelines. Cardiac output was calculated as the product of stroke volume and heart rate.

LV twist mechanics were quantified using speckle tracking technology as previously defined [14,26,27]. In brief, short-axis images of the LV base (proximal portion of the mitral valve leaflets visible in end-diastole) and of the LV apex (smallest circular image obtained when moving the transducer cranial from the apical 4-chamber view window) were obtained. Following determination of the best echocardiographic window at rest, the chest was marked up to serve as a quick guide to locate the best window during exercise. Additionally, the trained sonographer then adjusted the transducer angle minimally to obtain a similar image to that obtained at rest. For analysis, the endocardial border of short-axis images of the LV base and apex were manually traced by placing 12 equidistant markers across the 360 degrees’ image, and a region of interest was created that covered the entire contractile myocardium, excluding valves and trabeculations. Raw speckle tracking data generated by the software (EchoPAC Version 112 revision 1.0, GE Vingmed Ultrasound, Horten, Norway) were saved and exported into custom software (2D Strain Analysis Tool, Version 1.0beta14, Stuttgart, Germany). The custom software applied a generic cubic spline algorithm to interpolate the raw speckle tracking data to 600 data points in systole and diastole, respectively. In accordance with the principles outlined by Arts *et al*. [16], twist-to-shortening ratio (TSR) was calculated similar to the methods described in a recent publication [28]. Here, we multiplied the frame-by-frame basal and apical rotation data by their respective frame-by-frame circumferential strain (%) data obtained from the identical procedures outlined above. Then, apical frame-by-frame data were subtracted and the peak value was identified. This peak value was then multiplied by the maximal shortening (%) of the LV length from end-diastole to end-systole. All twist mechanics data referred to in this study are based on interpolated results and represent the average of the entire myocardium. For statistical analyses and reporting of results, the peak values in systole (LV twist) or early diastole (untwisting rate) were used.

#### Blood pressure and heart rate

Peak systolic, diastolic and mean arterial blood pressure were analysed from the recorded waveform and averaged over the last two minutes of exercise. Similarly, heart rate was determined by the intervals of the systolic blood pressure peak and averaged over the last two minutes of exercise.

#### Statistical analyses

For 14 of the dependent variables (DV), differences between means across the three exercise conditions were analysed using the one-way analysis of variance for repeated measures (ANOVA RM). For the remaining four DVs (Q, SV, EDV and ESV) missing values were present. As a consequence, in these cases, we were able to fit a general linear model (ANOVA GLM 4) to these data. In all cases, residuals and fits were saved when running the ANOVAs. All residuals were confirmed as being drawn from a population that was normally distributed on the variables of interest (Anderson-Darling test). In considering sphericity, homoscedastic (additive) error was confirmed in all cases by correlating (Pearson’s) absolute residuals against fitted vales. When main effects were identified as statistically significant (P ≤ 0.05), Tukey tests were applied as the post-hoc analysis for the ANOVA RMs, and paired samples t-tests, with a Dunn-Sidák correction to the level of statistical significance, were used as the post-hoc tests for the ANOVA GLM 4s. To determine whether LV twist mechanics responded more consistently to relative exercise intensity or IAT, the within-groups variance between exercise conditions was determined from the F-ratio of (individual mean squares (MS)/residual MS). Because we were confident in the likely direction of the outcome, we directionalized our hypotheses to test the alternative (*H*_*1*_: one-tailed) rather than the null (*H*_*0*_: two-tailed), for differences in LV twist mechanics during 40%peak compared with responses during 40%peak+HYP. These were estimated using a one-tailed paired samples t-test based upon the aforementioned hypothesis. Relationships between dependent variables were determined with reference to un-weighted ordinary least squares linear regression analysis. Statistical analyses were performed using GraphPad Prism (GraphPad Prism for Windows, Version 5.0.1, San Diego, California, USA) and Minitab v17 (Minitab Inc., State College, Philadelphia, USA). Statistical significance was accepted at P ≤ 0.05. Data are reported as means ± standard deviations unless otherwise highlighted.

## Results

**Fig 1:**
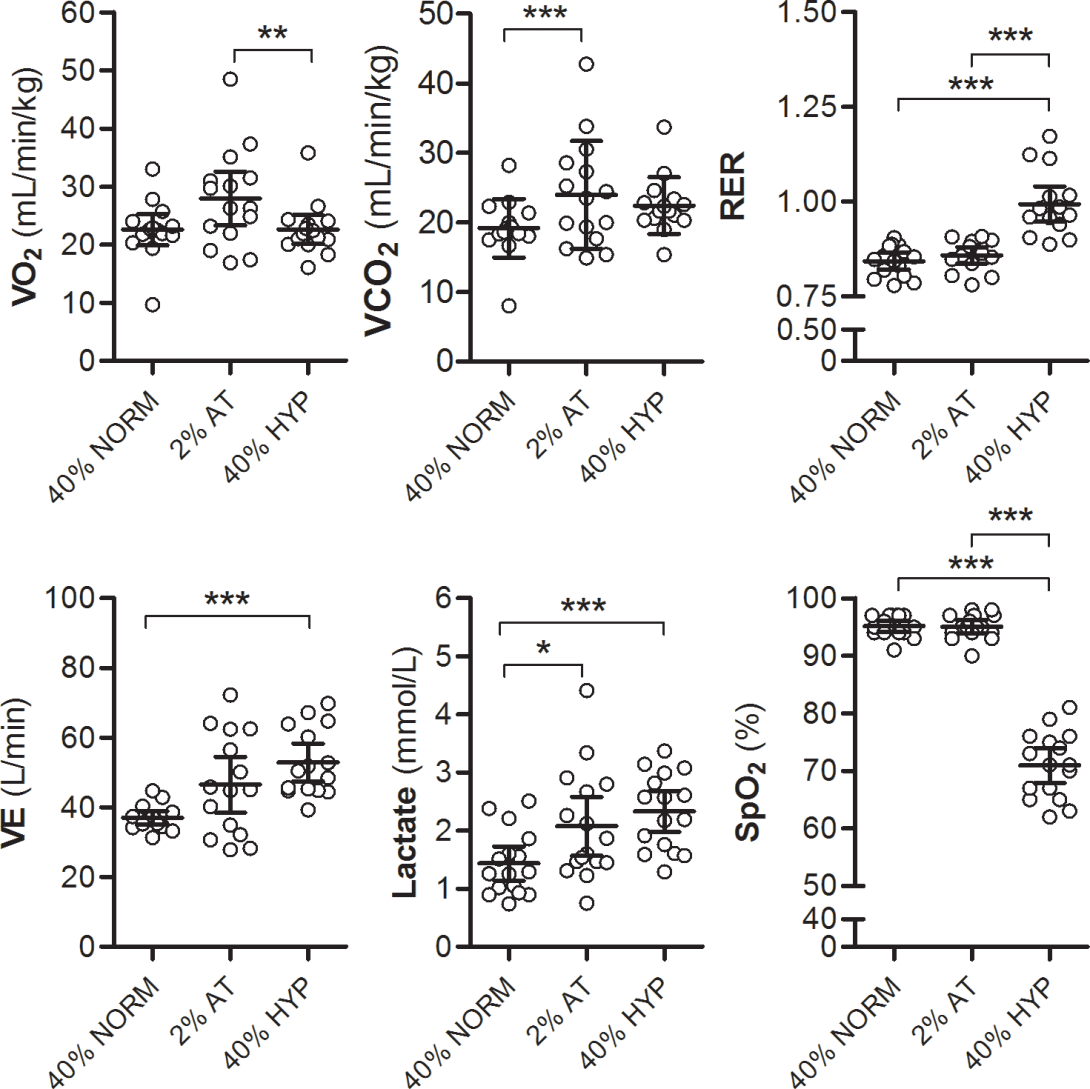
Metabolic responses during three exercise conditions. The lower arterial oxygen saturation (SpO_2_) during exercise at 40%_peak_ in hypoxia (40%peak+HYP) did not affect total whole-body oxygen consumption ($\dot{V}$O_2_) due to a compensatory increase in ventilation (VE). However, as a consequence of altered metabolism, lactate and RER were significantly increased during 40%peak+HYP. IAT = individual anaerobic threshold; *: *P* < 0.05; **: *P* < 0.01; ***: *P* < 0.001.

Summary results of all data are presented in Table 1. The mean power output did not differ significantly between exercise conditions (*P* > 0.05) although the variance was significantly greater during exercise at IAT + 2%, as per the study design (*F*_2,47_ = 2.16, *P* = 0.02). $\dot{V}$O_2_ did not differ statistically during 40%peak compared with 40%peak+HYP, however, it was significantly greater during exercise at IAT + 2% (*P* < 0.05). In contrast, VE and lactate were similar between exercise at 40%peak+HYP and exercise at IAT + 2% but were lower during 40%peak. SaO_2_ was significantly reduced during hypoxia only (*P* < 0.001), see Fig 1.

**Table 1:** General cardiovascular function and LV twist mechanics at rest and during three exercise conditions.

	Rest[99% CI]	40%_peak_/NORM[99% CI]	Exercise IAT + 2%[99% CI]	40%_peak_/HYP[99% CI]
Power output (W)	-	82±12	97±36	82±12
	-	[74to89]	[74to120]	[74to89]
SaO_2_ (%)	98±1	95±2	95±2	71±6***†††
	[97to98]	[94to96]	[94to96]	[67to75]
$\dot{\text{V}}$O_2_ (mL/min/kg)	6.0±1.2	22.6±4.9	27.9±8.3	22.6±4.4††
	[5.2to6.8]	[19.3to25.8]	[22.4to33.5]	[19.6to25.6]
$\dot{\text{V}}$CO_2_ (mL/min/kg)	4.9±1.2	19.2±4.2	24.0±7.7	22.4±4.1***
	[4.1to5.7]	[16.4to22.0]	[18.8to29.1]	[19.7to25.1]
RER	0.78±0.07	0.84±0.04	0.86±0.04	0.99±0.08***†††
	[0.73to0.83]	[0.82to0.87]	[0.83to0.88]	[0.94to1.05]
VE (L/min)	10.8±1.6	37.0±3.5	46.5±14.4	52.9±9.7***
	[9.8to11.8]	[34.6to39.4]	[37.3to55.8]	[46.6to59.2]
Lactate (mmol/L)	0.8±0.3	1.4±0.5	2.1±0.9*	2.3±0.6***
	[0.6to1.0]	[1.1to1.8]	[1.5to2.7]	[1.9to2.7]
Cardiac output (L/min)	4.8±1	9.4±2	10.4±1.8	11.2±1.8
	[4.1to5.4]	[8.1to10.7]	[9.1to11.6]	[10.2to12.4]
Heart rate (bpm)	63±8	111±18	122±20	137±17 ***††
	[58to68]	[100to123]	[109to135]	[127to148]
Stroke volume (mL)	77±16	85±15	85±10	81±11
	[66to87]	[76to95]	[78to92]	[74to88]
End-diastolic volume (mL)	170±26	164±26	158±22	151±20
	[153to187]	[148to181]	[144to173]	[138to164]
End-systolic volume (mL)	93±19	79±16	73±18	70±14
	[81to106]	[69to90]	[61to85]	[61to79]
TSR (deg)	0.31±0.21	0.73±0.32	0.92±0.56	0.89±0.49
	[0.17to0.45]	[0.53to0.94]	[0.55to1.28]	[0.58to1.21]
RPP (bpm.mmHg)	8930±1667	20395±3090	22093±3333	26491±3514 ***†††
	[7856to10003]	[18405to22385]	[19947to24239]	[24228to28754]
Systolic BP (mmHg)	141±12	185±14	183±19	193±21
	[133to149]	[175to194]	[171to195]	[180to207]
Diastolic BP (mmHg)	78±6	96±8	93±9	94±9
	[74to82]	[91to101]	[87to99]	[88to100]

AT: Anaerobic threshold; RPP: Rate pressure product; SaO_2_: arterial oxygen saturation; Systolic BP: Systolic blood pressure

*,**,***: *P* < 0.05, *P* < 0.01, *P* < 0.001 *vs*. normoxia

^††^, ^†††^: *P* < 0.01, *P* < 0.001 *vs*. IAT + 2%. *Note*: *Statistical comparison between conditions does not include resting data*.

**Fig 2:**
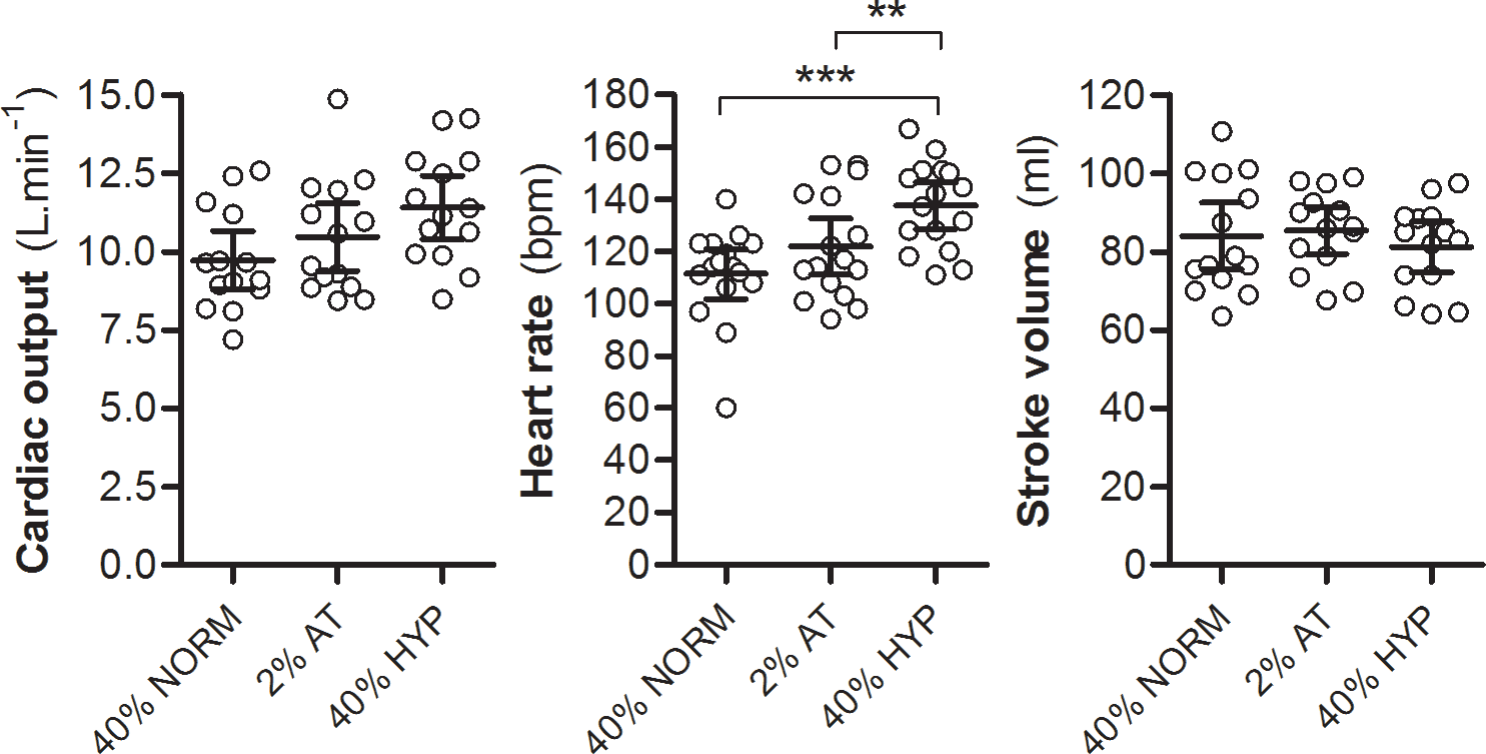
Cardiac output, heart rate and stroke volume. The increase in cardiac output during exercise in hypoxia (40%peak+HYP) was entirely attributed to an increase in heart rate, while stroke volume was not statistically different between exercise conditions, suggesting a purely autonomic and non-mechanical difference in hypoxia. IAT = individual anaerobic threshold; *: *P* < 0.05; **: *P* < 0.01; ***: *P* < 0.001.

The significantly higher cardiac output during 40%peak+HYP compared with 40%peak was entirely attributable to a higher heart rate because end-diastolic volume, end-systolic volume and stroke volume were not statistically different between exercise conditions (Fig 2). Systolic blood pressure was mildly elevated during exercise at 40%peak+HYP compared with exercise at IAT + 2% (*P* < 0.05), contributing to the higher rate pressure product, RPP, during 40%peak+HYP (*P* < 0.05).

**Fig 3:**
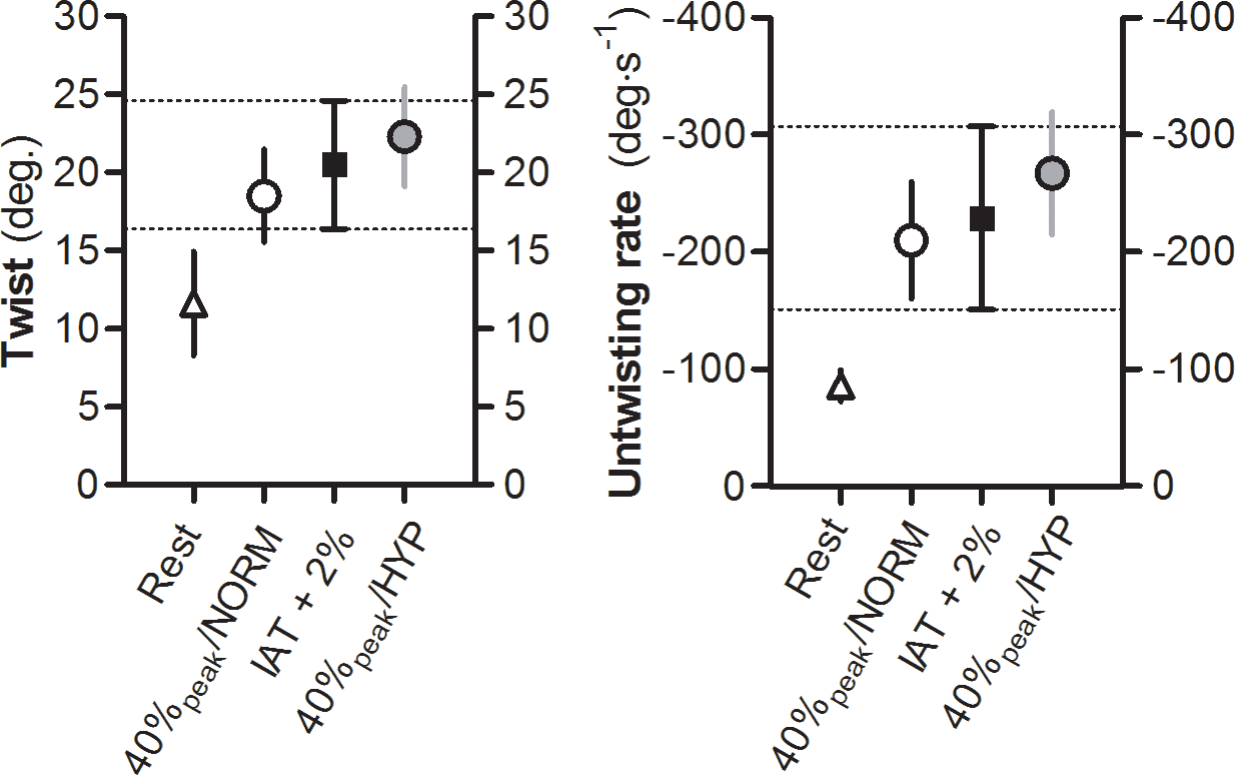
Variability of LV twist mechanics during three different exercise conditions. The graphs show the variability of LV twist and untwisting rate during three exercise conditions, performed by the same individuals. The variability of LV twist was significantly greater during exercise at 2% above individual anaerobic threshold (IAT + 2%), while the variability of LV untwisting rate was statistically not different between exercise conditions. Data are mean ± 99% CI.

**Fig 4:**
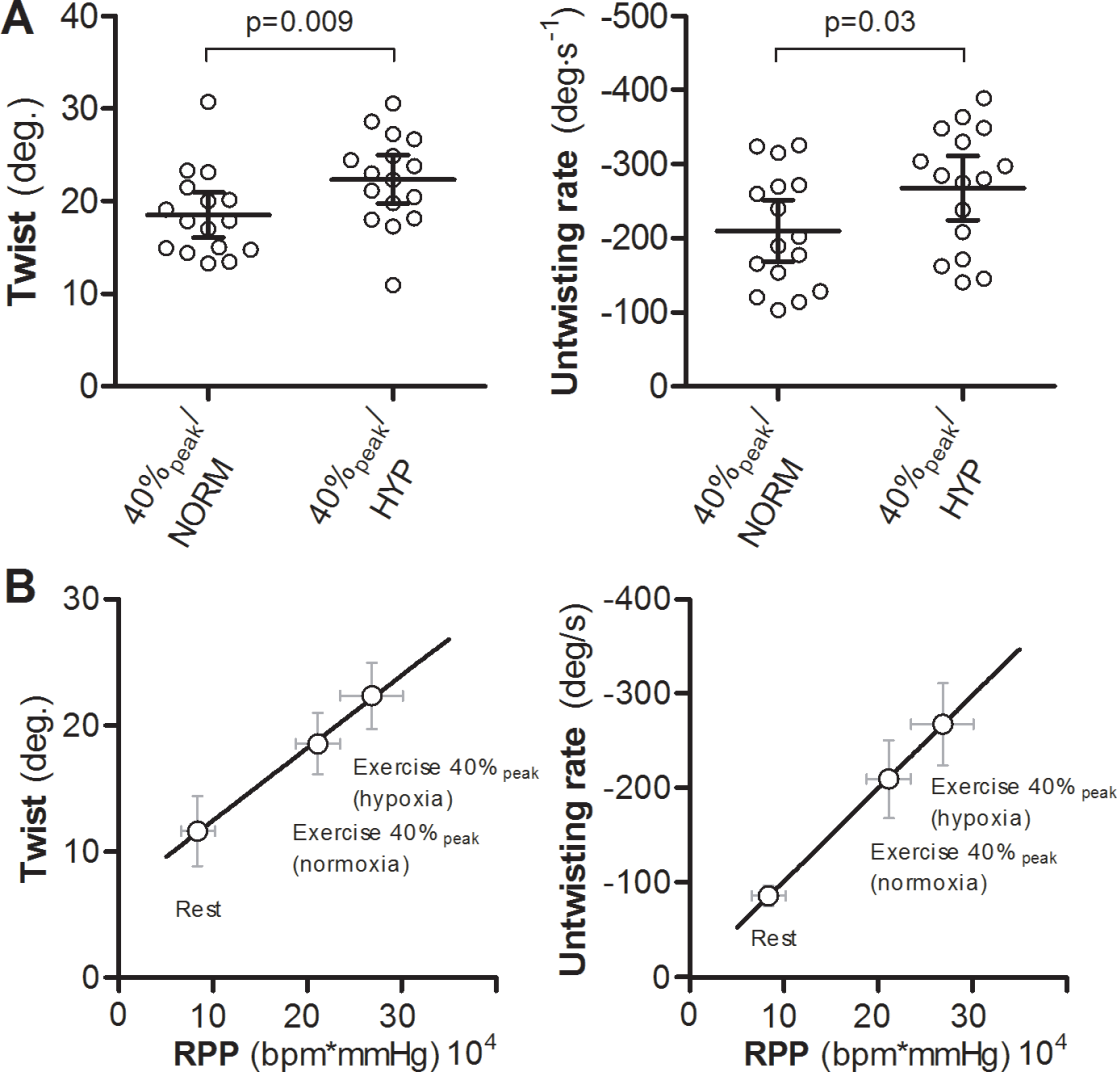
Effect of hypoxia on LV twist and untwisting rate during exercise. Despite the same whole-body oxygen consumption ($\dot{V}$O_2_) and LV volumes, hypoxia significantly increased LV twist mechanics (**A**), indicating that relative exercise intensity cannot be the sole determinant of the absolute magnitude of LV twist mechanics. However, when LV twist mechanics were plotted against rate pressure product (RPP), a clear linear relationship was observed, suggesting that myocardial work may ultimately predict the magnitude of LV twist and untwisting rate (**B**).

LV twist and untwisting rate were significantly higher during 40%peak+HYP compared with 40%peak. In relation to our main research question, the variance of LV twist was significantly greater during exercise at IAT + 2% (*F*_2,47_ = 2.08, *P* < 0.05). In contrast, the variability of LV untwisting rate was not statistically different between conditions (*F*_2,47_ = 1.172, *P* > 0.05, Fig 3 and Table 2). Overall, LV twist and untwisting rate were linearly related to RPP (Fig 4). TSR did not differ significantly between the three exercise conditions (*P* > 0.05).

**Table 2:** LV twist mechanics at rest and during three exercise conditions.

	Rest	40%_peak_/NORM	ExerciseAT + 2%	40%_peak_/HYP	*F*_2, 47_	*P-*value
**LV twist (degrees)**	11.6±5.2	18.5±4.6	21±6.3	22.3±4.9	2.08	0.04
[99% CI]	[8.2to14.9]	[15.5to21.5]	[16.4to24.6]	[19.1to25.5]		
Margin of error	3.35	3	4.1	3.2		
**LV UT rate (degrees/sec)**	-86±21	-210±78	-229±122	-267±82	1.17	0.34
[99% CI]	[-72to-100]	[-160to-260]	[-151to-307]	[-215to-320]		
Margin of error	14	50	78	52.5		

Data are mean ± SD. *F*_2, 47_ *= F-*ratio of individual mean squares / residual mean squares. *Note*: *Statistical comparison between conditions does not include resting data*.

## Discussion

### General cardiovascular responses to normoxic and hypoxic exercise

In the present study, the general cardiovascular responses to exercise in normoxia and hypoxia were in agreement with current opinion in exercise physiology. $\dot{V}$O_2_ did not differ significantly between 40%peak+HYP compared with 40%peak, which reflects a similar demand for O_2_ in both conditions. However, as per the study design, hypoxia was accompanied by a heterogeneous response in O_2_ desaturation, increased ventilation and cardiac output and enhanced blood lactate concentrations [29]. With regard to LV function, LV end-diastolic volumes and end-systolic volumes were similar in both 40%_peak_ exercise trials, which agrees with previous reports [30] and suggests that acute normobaric hypoxia did not influence preload of the heart. Consequently, this allows for the direct comparison between exercise at 40%peak *vs*. 40%peak+HYP independent of the influence of phenomena such as the Frank-Starling mechanism [31]. This finding is essential for the interpretation of LV twist mechanics as discussed in the next section.

### Variability and magnitude of LV twist mechanics

Although generic exercise responses have been studied extensively to date, the role of LV twist mechanics during exercise is relatively unexplored. To our knowledge, the first major report on LV twist mechanics during exercise was published less than a decade ago [7]. This and other studies [8,9,10] have provided insight into the link between mechanical myocardial performance and cardiac health and have, thus, indirectly highlighted the importance of measuring LV twist mechanics. Studies in healthy humans have confirmed the sensitivity of LV twist mechanics to respond to varied physiological stimuli, including exercise [3,4,5,6,13,32,33]. However, it was previously noted that LV twist responses are often characterised by a relatively large variability between individuals. The present study shows, for the first time, that some of the variability of LV twist mechanics during exercise might be related to the choice of exercise intensities that are prescribed for ‘stress testing’. Importantly, the present responses did not appear to be influenced by an altered loading state nor by the heart rate, as heart rate was less variable during hypoxia (99% CI margin of error = 11) than during IAT+2% (99% CI margin of error = 13). This finding is in accordance with a previous study showing that some parameters of cardiac deformation do not appear to be strictly coupled with heart rate [34]. The source of lower variance in LV twist mechanics during exercise at the same relative exercise intensity requires further investigation. Of the parameters investigated in the present study, the VO_2_ response during normoxia and hypoxia was considerably less variable (99% CI margin of error = 3.3 and 3.0, respectively) compared with that during IAT+2% (99% CI margin of error = 5.6), despite a clearly greater variability of SpO_2_ in hypoxia as intended by the study design (99% CI margin of error = 4.0) compared with normoxia or IAT+2% (99% CI margin of error = 1.0 and 1.0, respectively). Consequently, we propose that future studies should focus on the role of systemic O_2_ consumption in the determination of LV twist during exercise.

Although more laborious than using a single exercise bout prescribed by absolute workloads or a target heart rate [[7,9], respectively; Note that the approach of using a ‘target heart rate’ during exercise testing is also quite problematic because heart rate reserve, HRR, can differ significantly between individuals, as described in more detail in: [22]], the present study emphasises the need to perform a low risk [35] maximal exercise test that is used to then prescribe subsequent intensities by the same relative workload. The present study strongly supports incorporation of this approach in the increasingly popular exercise testing of patients for the assessment of dynamic cardiac deformation responses [7,9,36].

With regard to the magnitude of LV twist mechanics during exercise, we show that LV twist mechanics can be further influenced by acute factors that alter myocardial demand, as reflected here by RPP. To date, a direct relationship between LV twist mechanics and myocardial work during exercise does not appear to have been reported (it must be emphasised that RPP is not synonymous to myocardial oxygen consumption, but in the context of this study the authors believe that RPP is an acceptable approximation for the increased demand on the heart known to occur during hypoxia). The closest previous approximation that may relate to the present findings was provided by Beyar & Sideman [37], who showed that the known LV architecture and resultant mechanical “twisting motion” appear to be related to the O_2_ distribution across the LV muscle (‘transmural’). The calculations by Beyar & Sideman [37] support the present results which suggest an association between myocardial O_2_ demand and LV twist mechanics. Importantly, this may not reflect an altered contribution from endocardial and epicardial fibres as our TSR results showed no significant differences between exercise conditions. Rather, the present results may be associated with either an unequal distribution of fibre stress unrelated to heart muscle O_2_ demand or factors such as increased contractility. Although hypoxia causes prominent vasodilation, it is possible that it also results in sub-endocardial ischemia [38] and therefore adjustments in LV twist and untwisting rate may be required to restore the optimal distribution of O_2_ across the LV muscle [23,37]. The new finding might explain some of the remaining variability in the responses between individuals and may have implications for the interpretation of LV twist mechanics in research and the clinical setting. For instance, if LV twist mechanics are closely related to myocardial O_2_ demand, then any physiological processes occurring during normal daily living such as thermoregulation, dehydration and physical effort that acutely alter myocardial demand and impact LV twist mechanics [3,5,39,40] will further influence the magnitude of LV twist at rest and during exercise, even when exercise intensities are standardised. Interpreting the response of LV twist mechanics during exercise must therefore take into account the prevailing myocardial work at a given effort. Although a challenging task, this is an exciting new opportunity because it might prove essential in the understanding of the variability in LV twist mechanics between individuals.

## Conclusions

In healthy humans, the variability of LV twist mechanics during exercise is significantly lower at the same relative exercise intensity compared with exercise at anaerobic threshold. Moreover, the magnitude of LV twist mechanics is further influenced by hypoxia and LV twist mechanics are related to rate pressure product. Together, the data suggest that diagnostic exercise tests in clinical and research settings must be standardised by prescribing relative exercise intensities and the interpretation of LV twist mechanics must take into account the prevailing myocardial work.
